# Radiological Discovery of Asymptomatic Megaureter: A Rare Urological Anomaly and Literature Review

**DOI:** 10.7759/cureus.93895

**Published:** 2025-10-05

**Authors:** Oluseyi H Ogunfusika, Ayodeji O Adewoye

**Affiliations:** 1 Urology, Kettering General Hospital NHS Foundation Trust, Kettering, GBR; 2 Accident and Emergency, Kettering General Hospital NHS Foundation Trust, Kettering, GBR

**Keywords:** congenital mega ureter, hydonephrosis, lower urinary tract symptoms (luts), mega ureter, primary obstructive megaureter, robotic ureteric re implantation, unilateral vesicoureteral reflux, urolithiasis causing factors

## Abstract

Megaureter, characterised by a dilated ureter without evidence of obstruction at the vesicoureteral junction, is a condition primarily associated with paediatric populations. It is often diagnosed early in life due to urinary tract infections, hydronephrosis, or other urinary symptoms. In contrast, megaureters in adults are rarely encountered, particularly in asymptomatic individuals. This discrepancy in age-related presentation raises important questions regarding the natural history, detection, and clinical relevance of the condition in older patients.

This paper presents a rare case of an incidentally discovered, asymptomatic megaureter in an 80-year-old adult female who underwent abdominal imaging for new, declining renal function. Radiologic evaluation revealed bilateral markedly dilated ureters, right more than the left. The patient reported no history of urinary tract infections, flank pain, haematuria, or voiding difficulties. A comprehensive workup ruled out secondary causes, such as obstructive uropathy, neurogenic bladder, or ureteral stricture, supporting a diagnosis of megaureter.

In paediatric practice, primary megaureters are well-documented. Many are identified via prenatal ultrasound or early postnatal imaging and may require surgical intervention depending on the degree of dilation and associated complications. Conversely, the literature on adult megaureters, especially asymptomatic cases, is extremely limited, with only a handful of case reports and small series available. The condition may remain clinically silent for decades, only to be discovered incidentally during imaging for unrelated conditions, as in our case.

While intervention is often unnecessary in asymptomatic individuals with preserved renal function, proper identification is important to guide follow-up, prevent potential complications and distinguish benign congenital anomalies from acquired pathologies such as malignancy or obstructive uropathy. Surgical interventions, such as reimplantation of the ureter and endoscopic balloon dilatation, are reserved for some cases.

## Introduction

The ureter is a muscular, cylindrical structure that transports urine from the renal pelvis into the bladder. While its length varies slightly, a normal diameter is less than 3 mm [1]. While no specified limit exists, a megaureter is often considered as a ureteric size greater than normal. A study by Dekirmendjian et al. described a megaureter with a dilation of the ureter ≥ 7 mm [2]. The term megaureter is often used interchangeably with congenital or primary obstructive megaureter. These are better used as descriptive types of megaureters rather than describing the entire spectrum [3]. It remains largely a condition in children and is rare in adults. The majority of cases in children are spontaneously resolved. For conservative management, approximately a fourth of the cases will require surgical intervention. Conversely, the literature on adult megaureters, especially asymptomatic cases, is extremely limited, with only a handful of case reports and small series available. The condition may remain clinically silent for decades, only to be discovered incidentally during imaging for unrelated conditions, as in our case, or due to new declining renal function. In 1977, Smith et al. introduced the most comprehensive classification termed 'The international classification for megaureters'. They categorised the megaureters based on the presence or absence of reflux and/or obstruction at the ureterovesical junction into Obstructed, Refluxing, Unobstructed and None; each one of these was further classified as either primary or secondary [4]. Another practical classification was done by King in 1980, who categorised megaureters into primary obstructed (non-refluxing) megaureter (POM) and refluxing (non-obstructed) megaureter (dilating vesicoureteral reflux), as well as the two less common groups of obstructed refluxing megaureter and non-obstructive non-refluxing megaureter [5]. Megaureter is an imaging finding; hence, antenatal ultrasonography is usually the diagnostic tool in prenatal settings. For adults, ultrasound, computerised tomography (CT), or magnetic resonance Imaging (MRI) may be employed in the diagnosis. In addition, Mag 3 Renogram (mercaptoacetyltriglycine) or DMSA (dimercaptosuccinic acid) scans could also be a useful tool to determine differential kidney functions. These investigations are usually indicated in symptomatic patients being investigated for haematuria, urolithiasis, recurrent urinary tract infections (UTI) and worsening renal function. While many experts have suggested a possible aetiopathophysiology of this entity, a central explanation is that primary megaureter involves an aperistaltic portion of the lower ureter, which leads to functional obstruction and dilation, while the secondary megaureter, on the other hand, indicates obstruction or reflux downstream [6].

Our report emphasises the need for greater awareness of this clinical entity among adult care providers, particularly radiologists and urologists. Some hypotheses may explain the underreporting of adult asymptomatic megaureters. One of such possibilities is the spontaneous resolution or stabilisation of mild congenital ureteral dilation during childhood, leading to minimal clinical impact in adulthood. Alternatively, some megaureters may persist without progression or complications, evading detection unless imaging is pursued for other reasons. This raises the issue of whether current imaging protocols overlook such anomalies or whether these cases are simply underrecognized due to their silent course.

## Case presentation

**Figure 1:**
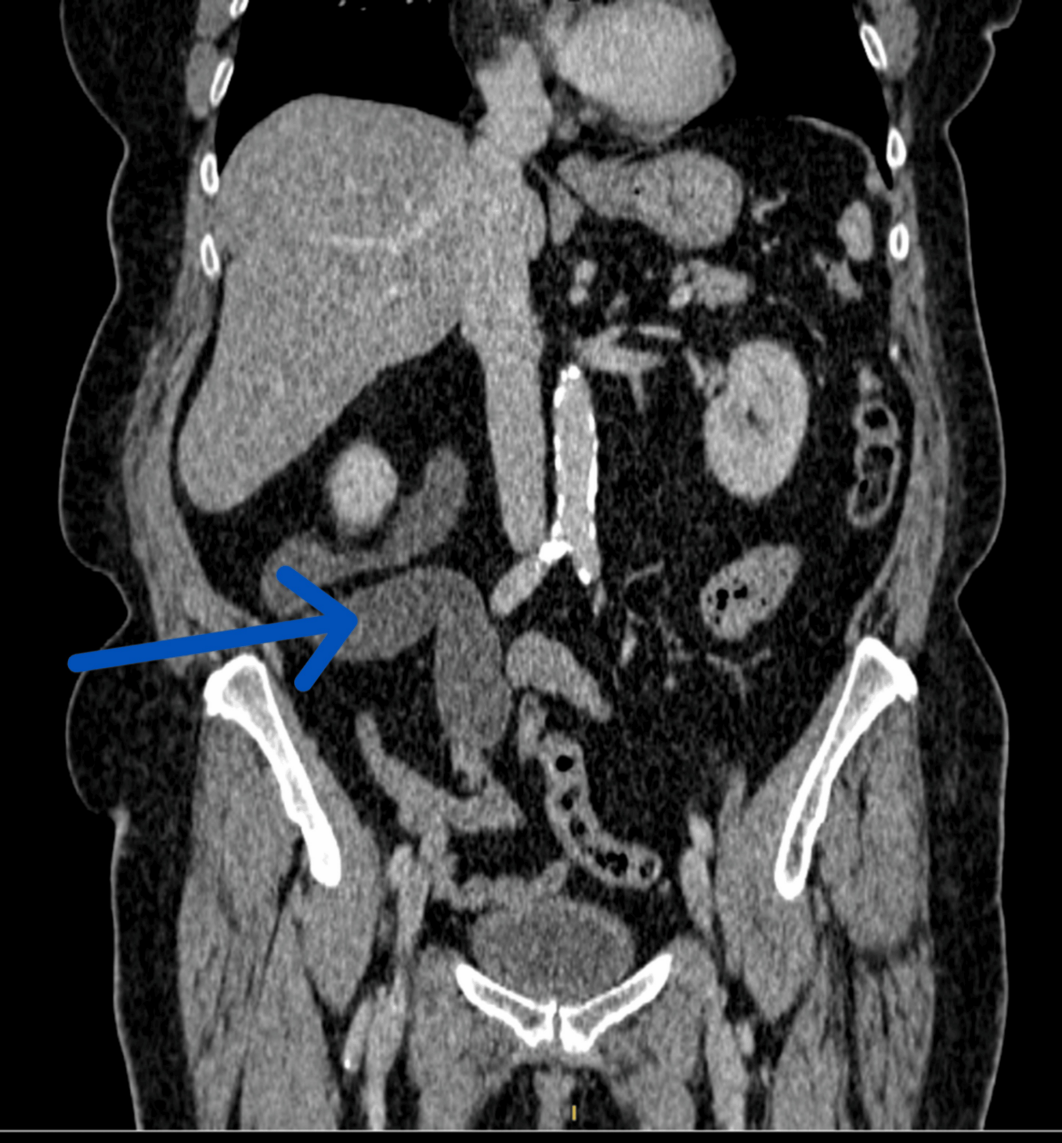
CT coronal imaging done eight years prior to attending the HOS (Haematuria One Stop) Urology Clinic The blue arrow shows the tortuous dilated right ureter.

An 80-year-old female, with no occupational risk for urothelial malignancy, was reviewed in the Haematuria One Stop (HOS) Urology Clinic on account of declining renal function, She was an ex-smoker, known to have a dilated ureter for over 8 years, a type 2 diabetic, hypertensive, and had a previous mastectomy for breast cancer and a right hemicolectomy (on yearly CT follow-up; Figure 1). She reported no abdominal pain or any other concerns. Physical and systemic examination revealed no significant findings; her post-void bladder scan was not significant at 100 ml. She had routine investigations, which included renal function profile (Table 1); for the electrolyte panel, a notable finding was the reduced estimated glomerular filtration rate eGFR).

**Table 1:** Electrolyte results in the HOS (Haematuria One Stop) Urology Clinic showing declining renal function eGFR: estimated glomerular filtration rate

Components – Electrolytes	Present	4 months prior to HOS	Range	Unit
Sodium	140	140	133 - 146	mmol/l
Potassium	5.5	4.4	3.5 - 5.3	mmol/l
Urea	6.3	4.1	2.9 - 8.2	mmol/l
Creatinine	101	62	60 - 120	umol/l
eGFR	45.3	85.6		

**Figure 2:**
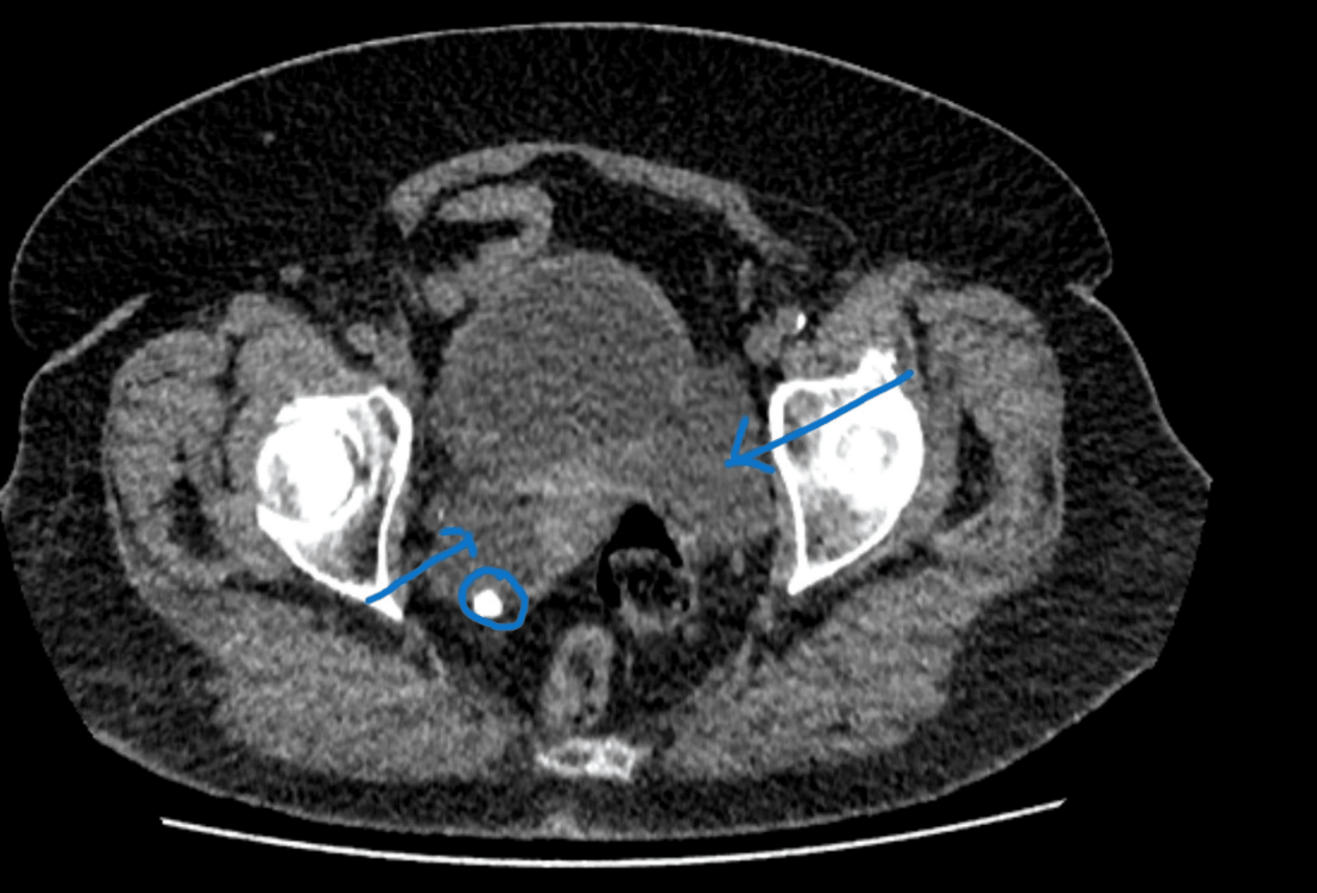
CT axial imaging showing bilateral dilated distal ureters (blue arrows) The right ureter contains the radiopaque stone.

CT urinary tract without contrast revealed grossly dilated ureters and worsening megaureters (Figure 2).

**Figure 3:**
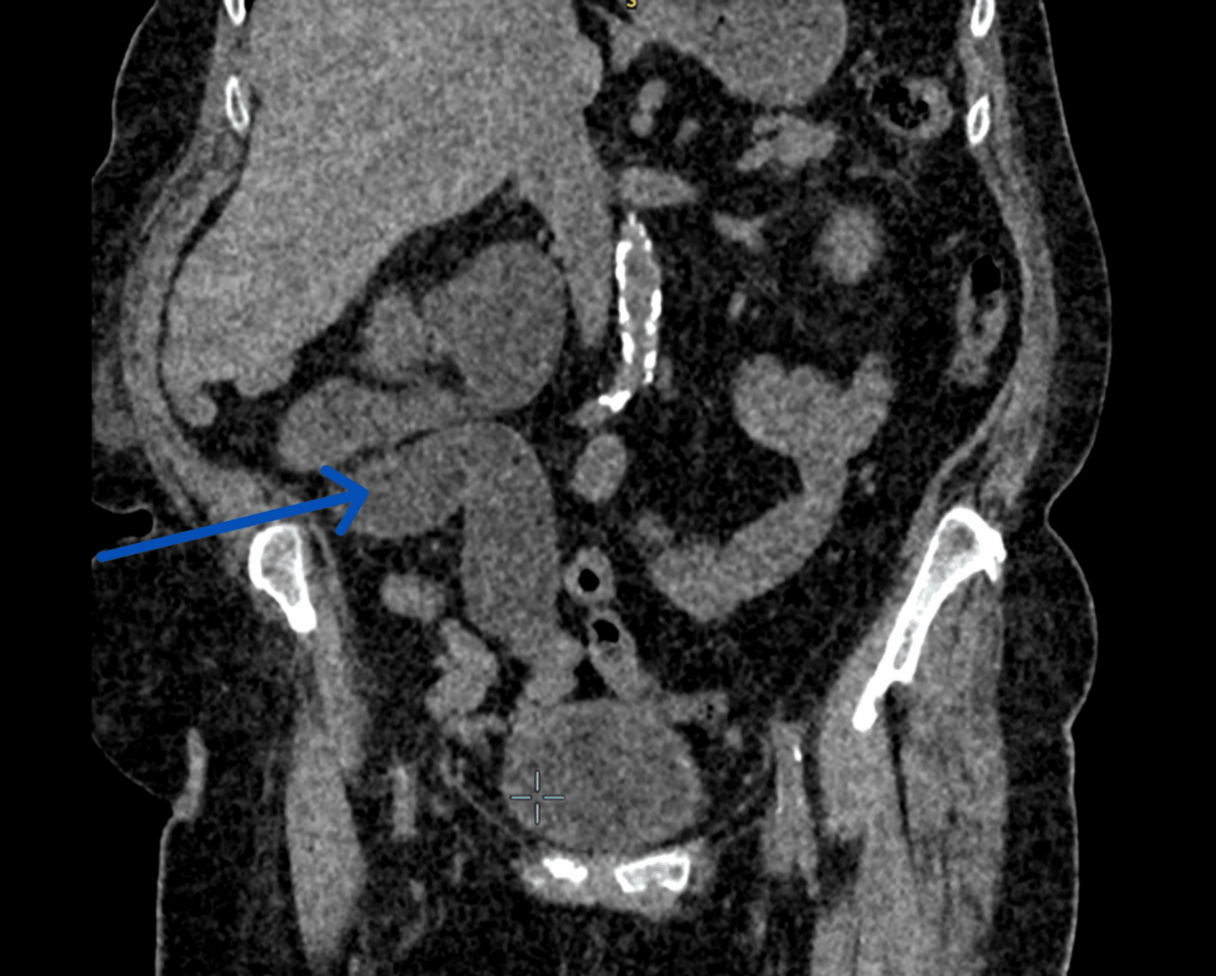
New CT coronal section The blue arrow shows a worsening, tortuous, dilated right ureter.

A new CT from the HOS shows worsening right hydroureter (Figure 3).

**Figure 4:**
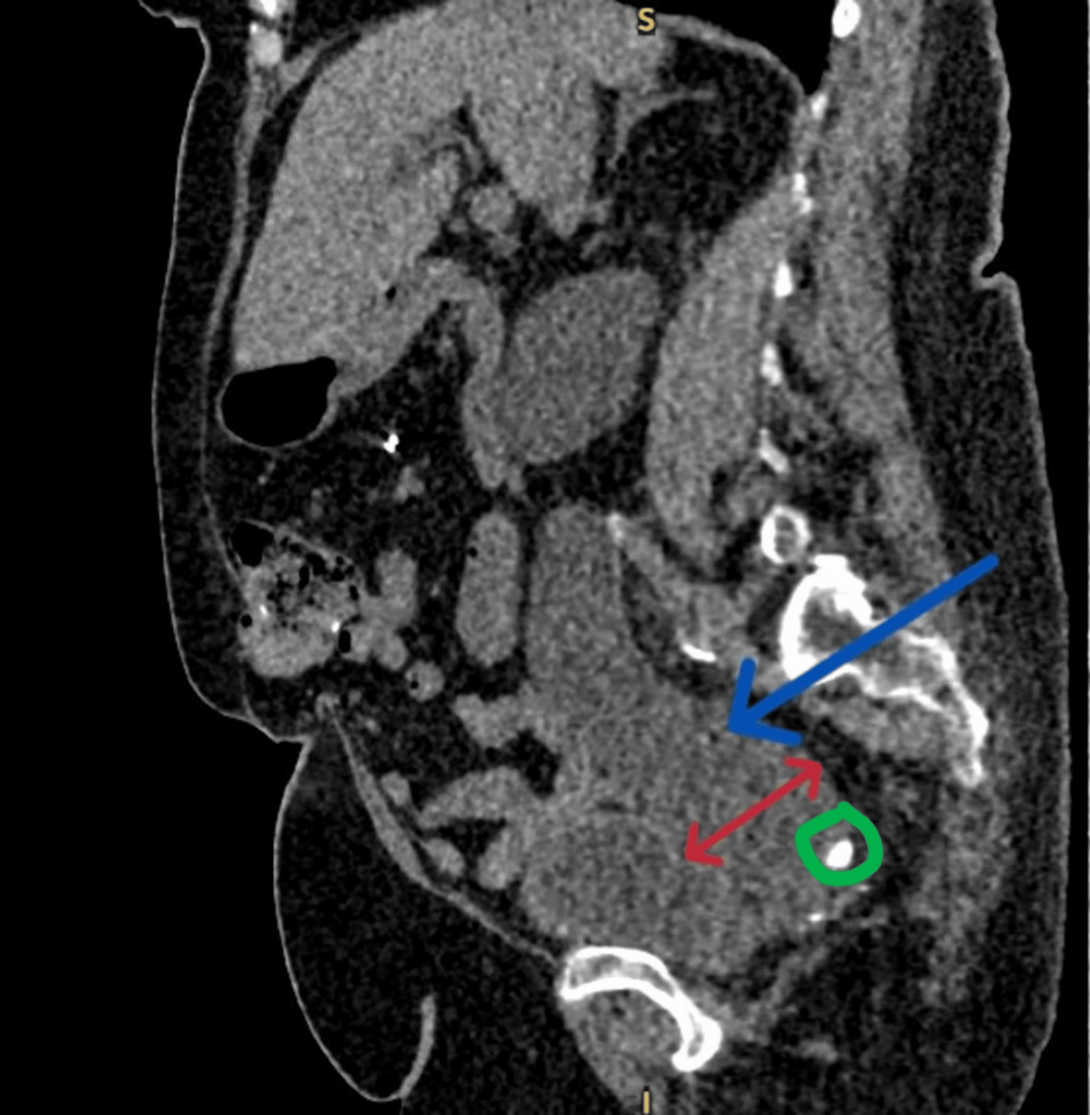
CT sagittal imaging showing a stone in the right distal ureter The blue arrow shows a dilated ureter, while the red arrow indicates the widest ureteric diameter measuring 5.26 cm. The green circle shows the radiopaque stone.

The CT shows a radiopaque stone in the right distal ureter (Figure 4).

**Figure 5:**
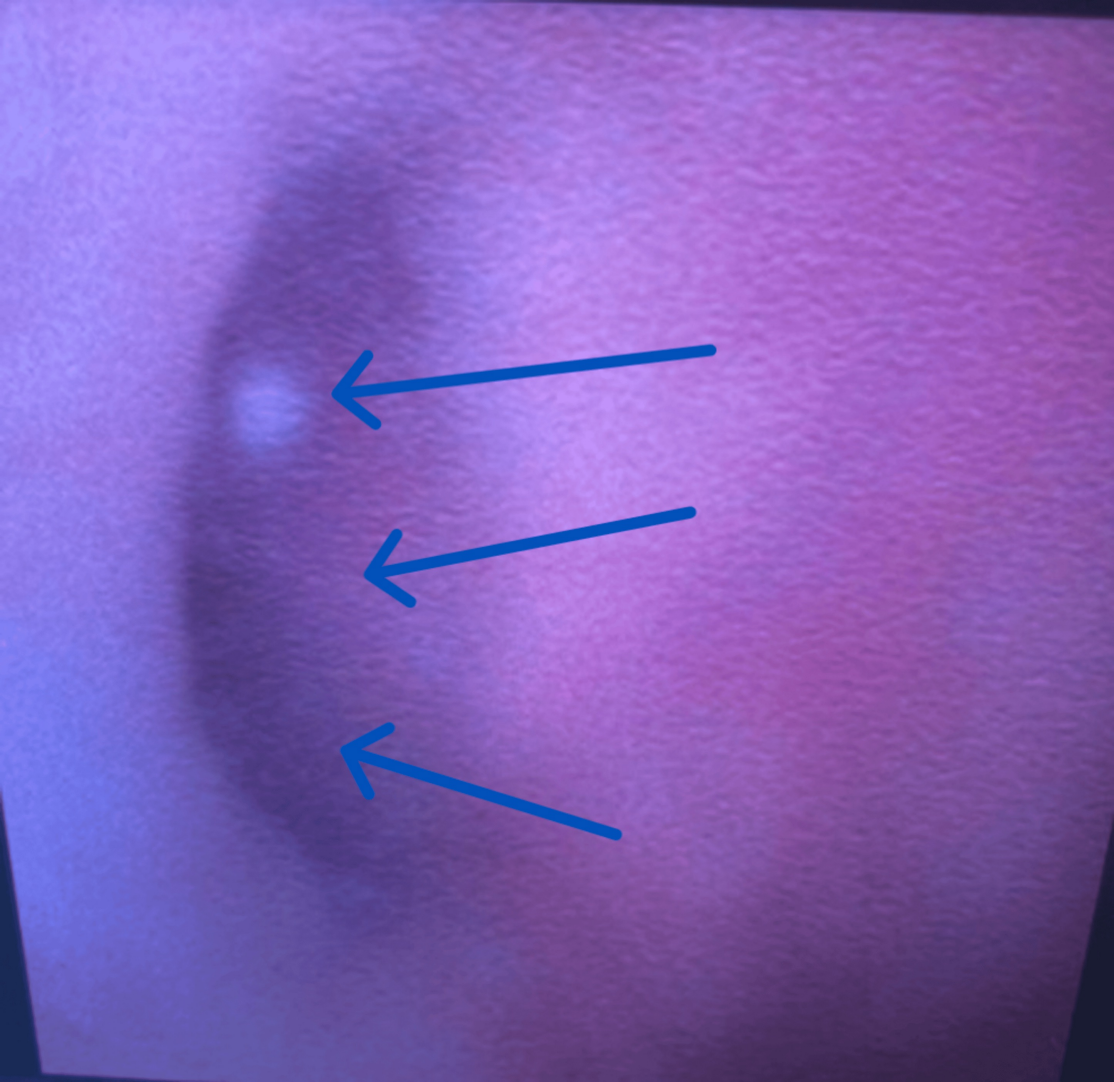
Flexible cystoscopy in the HOS (Haematuria One Stop) Urology Clinic showing a wide-open right ureteric orifice Blue arrows showing the ureteric orifice

Flexible cystoscopy in the HOS Urology Clinic revealed debris in the bladder, with no outright abnormal urothelial lesions and wide-open ureteric orifices (Figure 5).

The patient was discussed in the Urology multidisciplinary team (MDT) and subsequently offered a primary ureteroscopy for the small distal ureteric stone.

## Discussion

Megaureters are the second most likely cause of pathological neonatal hydronephrosis. They occur more often in males and are more likely to occur on the left side [6]. The natural history of asymptomatic POM is still somewhat of a mystery, especially in adults, as there are no well-documented cases in the literature to describe its full natural history [7]. Di Renzo et al. suggested that non-refluxing megaureters can stay stable without any treatment, while others warn that complications like infections, stone formation, and renal impairment can develop over time [8]. This was the case in our patient, who had been asymptomatic except for new, declining renal function.

The most prominent causes of primary obstruction are usually linked to intrinsic factors, such as a long, narrow, or atonic distal segment of the ureter; it could also be secondary, which is related to extrinsic factors like compression from surrounding structures. POM has also been linked to congenital abnormalities such as trisomy [9]. This obstruction increases the pressure within the ureter, resulting in hydroureteronephrosis and potentially affecting renal function. Over time, this can lead to urinary stasis, creating an environment that encourages urolithiasis [10]. The British Association of Paediatric Urologists surmised that primary megaureters should be conservatively managed at first. Symptoms such as pain or feverish urinary tract infections, as well as a differential renal function of less than 40% in asymptomatic patients linked to large or progressive hydronephrosis or a decline in differential function on serial renograms, are indications for surgical intervention [11]. While no clear surgical consensus exists in adults, patients with worsening pain, renal function, urolithiasis and recurrent UTI may require surgical intervention.

Surgical management of megaureter has evolved over the years. Historically, the gold standard for POM has been ureteral implantation with or without tapering (a 90-95% success rate); this is usually carried out through the open, laparoscopic, or robotic-assisted routes. Generally, this option poses a serious risk of potential complications arising from reimplanting a large ureter into a small bladder [12]. Therefore, endoscopic treatments have been proposed as alternative options in the initial management of POM, becoming popular in the last few years. Endoscopic management appears to be a definite alternative to reimplantation for a POM with a narrowed segment shorter than 3 cm [13]. Ortiz et al., in the largest study on POM reported in the literature, revealed a success rate of 87% for endoscopic balloon dilation; follow-up did not reveal any significant difference when compared to patients who underwent reimplantation [14]. For adults with a non-functioning kidney, scarred kidney or evidence of non-function confirmed on Mag 3 Renogram, a suitable option may be a nephroureterectomy as reported by Osaka et al. [15].

## Conclusions

Adult asymptomatic megaureters are a rare but clinically significant condition. The mainstay of management is to individualise assessment, evaluate renal function, anatomical development, and assess potential complications such as urolithiasis and recurrent UTIs. For effective treatment and to expand our knowledge of congenital urinary tract abnormalities outside of the paediatric population, this condition must be identified. Future research on the various surgical interventions is required.
